# Supplementary material for: Splice-Junction-Based Mapping of Alternative Isoforms in the Human Proteome
Source: Cell Rep. Author manuscript; Available in PMC 2020 Jan 15. (PMC6961840; doi:10.1016/j.celrep.2019.11.026)
Supplement: 2 [file NIHMS1546469-supplement-2.zip › DF1/PXD010154/Liver/Liver_73_PCK1_ADGFTLR.pdf]

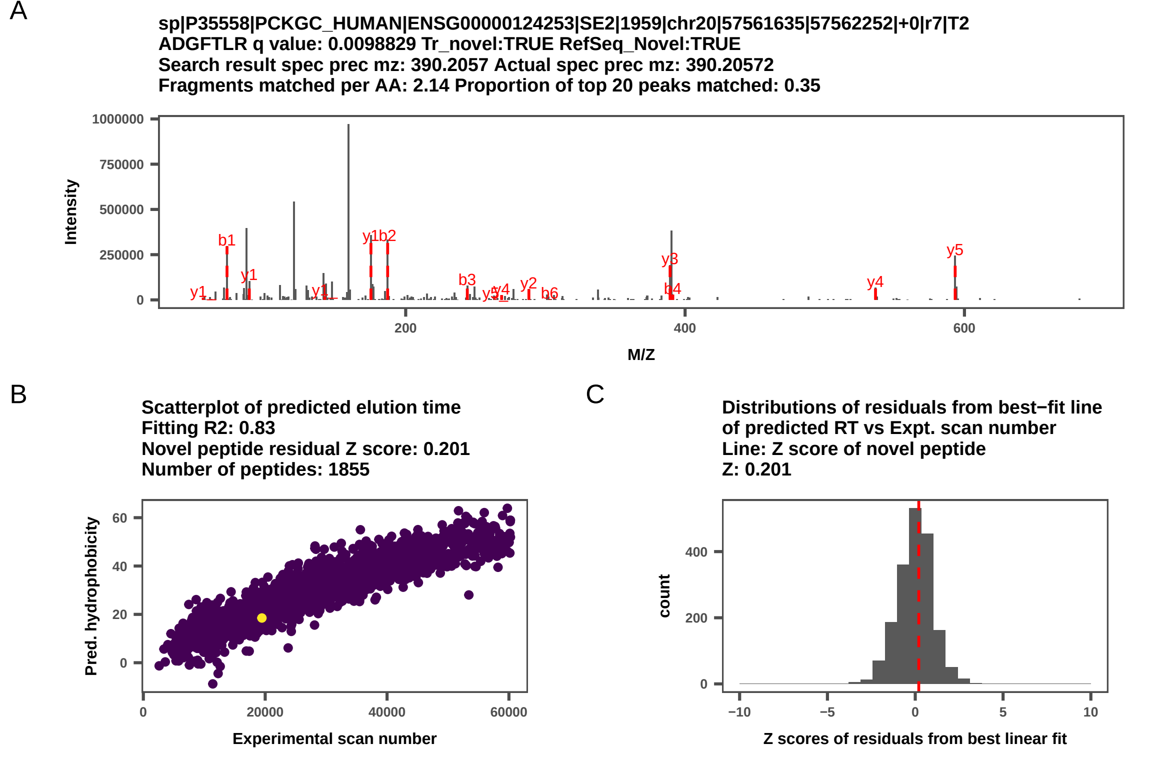

A
sp|P35558|PCKGC_HUMAN|ENSG00000124253|SE2|1959|chr20|57561635|57562252|+0|r7|T2
ADGFTLR q value: 0.0098829 Tr_novel:TRUE RefSeq_Novel:TRUE
Search result spec prec mz: 390.2057 Actual spec prec mz: 390.20572
Fragments matched per AA: 2.14 Proportion of top 20 peaks matched: 0.35
Intensity
1000000
750000
500000
250000
0
M/Z
200
400
600
b1
y1
y1
y1
y1b2
b3
y2
b6
y3
b4
y4
y5
B
Scatterplot of predicted elution time
Fitting R2: 0.83
Novel peptide residual Z score: 0.201
Number of peptides: 1855
Pred. hydrophobicity
60
40
20
0
Experimental scan number
0
20000
40000
60000
C
Distributions of residuals from best−fit line
of predicted RT vs Expt. scan number
Line: Z score of novel peptide
Z: 0.201
count
400
200
0
Z scores of residuals from best linear fit
−10
−5
0
5
10
